# Cholic Acid Protects In Vitro Neurovascular Units against Oxygen and Glucose Deprivation-Induced Injury through the BDNF-TrkB Signaling Pathway

**DOI:** 10.1155/2020/1201624

**Published:** 2020-10-10

**Authors:** Changxiang Li, Xueqian Wang, Juntang Yan, Fafeng Cheng, Xiaona Ma, Congai Chen, Wei Wang, Qingguo Wang

**Affiliations:** School of Traditional Chinese Medicine Department, Beijing University of Chinese Medicine, 11 Beisanhuandong Road, Chaoyang District, Beijing 100029, China

## Abstract

Ischemic stroke (IS) can disrupt various types of brain cells in the neurovascular unit (NVU) at both the structural and functional levels. Therefore, NVU is considered to be a more comprehensive target for the treatment of IS. It is necessary to develop drugs which targeted multiple mechanisms and cell types on NVU against IS. As a component of bile acid, cholic acid has been reported to be able to diffuse across phospholipid bilayers and further cross the blood-brain barrier (BBB). However, the effects exerted by cholic acid (CA) on the NVU after stroke remain unclear. Based on our previous research, we established and further supplemented the characteristics of the functional in vitro NVU model and its oxygen-glucose deprivation and reoxygenation (OGD/R) model. Then, we investigated the effect of CA on the maintenance of the in vitro NVU after OGD/R and further discussed the specific molecular targets that CA played a role in. For the first time, we found that CA significantly maintained BBB integrity, downregulated apoptosis, and mitigated oxidative stress and inflammation damage after OGD/R. Meanwhile, CA obviously increased the levels of brain-derived neurotrophic factor (BDNF), which were mainly secreted from astrocytes, in the coculture system after OGD/R. The results demonstrated that CA significantly increased the expression of TrkB, PI3K/Akt, MAPK/Erk, and CREB in neurons. These positive effects on the downstream proteins of BDNF were suppressed by treatment with ANA12 which is an inhibitor of TrkB. In conclusion, the present study demonstrates that CA exerted multiple protective effects on the NVU, mediated by increasing the release of BDNF and further stimulating the BDNF-TrkB-PI3K/Akt and BDNF-TrkB-MAPK/Erk signaling pathways in the context of OGD/R-induced injury. These findings indicate that CA possesses the effect of antagonizing multiple mechanisms of IS and protecting multiple cell types in NVU and may be useful as a treatment for IS.

## 1. Introduction

Stroke remains one of the leading causes of death and disability worldwide; IS accounts for over 80% of strokes [1]. The thrombolytic approach was the FDA-approved therapy for cerebral ischemia; however, only approximately 5% of patients benefit from this treatment [2]. In recent decades, many studies have been devoted to demonstrating that neuroprotective and neurorestorative therapies limit ischemic injury after stroke by promoting structural and functional recovery [3]. However, a majority of agents targeted a single event in the ischemic cascade and targeted the single neural element; the overspecifications may in part explain failure in the clinical setting [4]. It is strongly suggested that stroke be approached with multiple, multifaceted neuroprotective and neurorestorative methods. Therefore, the establishment of structural and functional units of organs in vitro is necessary to further understand brain functions and facilitate new treating approaches to enable cost-effective and more accurate predictions of drug efficacy [4].

NVU is a complex, integrated functional unit composed of neurons and neural supporting cells, such as astrocytes, as well as cells that comprise the vascular system including endothelial cells, pericytes, smooth muscle cells, and a brain-specific extracellular matrix [5]. Each cell type in the NVU plays an essential role, either in transmitting and processing neural signals or in maintaining the appropriate microenvironmental conditions for healthy neural function [5, 6]. The occurrence of IS can destroy NVU at both the structural and functional levels. The concept of NVU as a more comprehensive target for stroke treatment has emerged, and it has been reported that in vitro NVU culture models recapitulate brain-specific functions. Moreover, a major advantage of the in vitro NVU model over an in vivo model is the greater experimental control over the cellular and molecular interactions being investigated [7, 8].

Previous studies have reported in vitro NVU models using Transwell platforms, microfluidic platforms, brain slice, and other forms that were composed of endothelial cells, astrocytes, and neurons to form a structure that is closer to the function of brain tissue in vivo [9–11]. In the classical form of the in vitro NVU establishment, a number of groups have used Transwell platforms, and data obtained from their studies have provided seminal work to the field, highlighting the multicellular signaling pathways, the multitargeted therapeutic effects of drugs, and the effects of drugs passing the BBB on neurons underlying NVU [12, 13]. As the most common and widely used in vitro BBB model, the Transwell platform can be used to study the diffusion of drugs and the therapeutic effects of drugs across the BBB on neurons [14]. The BBB, a tissue site widely distributed in the cerebrum, is one of the NVU components and a specific biological barrier system, restraining and controlling the transport of endogenous and exogenous substances into the brain. The central nervous system is protected by the BBB [15]. Therefore, in order to more closely mimic the cerebral vasculature and cortex, a more complex in vitro system, in which brain microvascular endothelial cells (BMECs) were cultured with astrocytes and neurons, was established and used to evaluate new drugs against neurological diseases. We observed the protective effect of hyodeoxycholic acid on in vitro NVU which was constructed by planting astrocytes, BMECs, and neurons in Transwell platforms [16]. In addition, we constructed an in vitro BBB using astrocytes and BMECs and observed the protective effect of geniposide on it [17].

CA as one of the components of bile acid has been reported to be able to diffuse in the phospholipid bilayer and further cross the blood-brain barrier [18]. Meanwhile, CA as the main active ingredient of bezoar has been exhibited to possess neuroprotective effects [19, 20]. In our previous study, CA combined with hyodeoxycholic acid, baicalin, and geniposide prevented ischemia-induced brain injury with a time window of 6 h [21].

BDNF, the most widely distributed and extensively studied neurotrophins, exert a neuroprotective effect against ischemic brain injury and are the most important signaling molecules for adaptive brain plasticity after stroke [22]. BDNF interact with tropomyosin-related kinase receptor type B (TrkB), which subsequently activates the PI3K/Akt and MAPK/Erk signaling pathways with various functions including prosurvival activity, antiapoptosis, anti-inflammation, and enhancement of dendritic growth and branching [23].

Here, we established a functional NVU by the coculture of neurons, astrocytes, and BMECs, aiming to mimic brain tissue, and analyzed the BBB function and neuronal morphological phenotype of the NVU model under normal and OGD/R condition. For the first time, we investigated the potential protective effects exerted by CA on the NVU system using an OGD (1 h)/R (24 h) model. The effects of CA on the blood-brain barrier (BBB) integrity, apoptosis, oxidative stress, inflammation, BDNF levels, and expression of TrkB, PI3K, Akt, MAPK, and Erk were measured. We hypothesized that CA produces neuroprotection on NVU against ischemia through the BDNF-TrkB-PI3K/Akt signaling pathway and the BDNF-TrkB-MAPK/Erk signaling pathway.

## 2. Materials and Methods

### 2.1. Animals

Newborn Sprague-Dawley (SD) rats of indicated days were purchased from Beijing Weitong Lihua Experimental Animal Technology (license no. SCXK 20160006, Beijing, China). Animal welfare and experimental procedures were carried out in accordance with the National Institutes of Health *Guide for the Care and Use of Laboratory Animals* and were approved by the Ethics Committee of Experimental Animals of Beijing University of Chinese Medicine (BUCM-3-2016040201-2003).

### 2.2. Isolation and Culture of Primary Neurons

Primary neurons were obtained as our previous study [16]. Brains were dissected from 0 to 24 h old newborn SD rats. The brains were cut sagittally into halves, and the meninges were removed with tweezers. The cortices were dissected away, and much of the white matter was removed. The cortex was digested at 37°C for 20 min with 0.125% trypsin-EDTA (Sigma-Aldrich, St. Louis, MO, USA). The cortical tissues were homogenized to single cell suspensions with medium containing 10% fetal bovine serum (FBS, Gibco-BRL, Grand Island, NY, USA). Then, the suspension was filtered through a 70 *μ*m cell strainer and centrifuged at 800 rpm for 5 min. The precipitate was resuspended with Neurobasal-A medium (Gibco-BRL) containing 10% FBS, 2% B27 (Invitrogen; Thermo Fisher Scientific, Inc.), 0.25% GlutaMAX (Gibco-BRL), and 1% penicillin/streptomycin (P/S, Gibco-BRL). Cells were then seeded at a density of 1 × 10^6^ cells/mL into 0.01% poly-L-lysine (PLL, Sigma-Aldrich) precoated dishes at 37°C and 5% CO_2_. The medium was changed to nonserum formula after seeding for 4 h, and 50% of the medium was replaced every 2 days.

### 2.3. Isolation and Culture of Primary Astrocytes

Primary astrocytes were obtained as our previous study [17]. Cerebral astrocytes were obtained from brain cortices of 2-3-day-old SD rats. Briefly, after removal of meninges and white matter, the cerebral cortex was digested first with 0.125% trypsin-EDTA at 37°C for 10 min and transferred into Dulbecco's modified Eagle's medium (DMEM)/F12 with 10% FBS and 1% penicillin/streptomycin. The suspension was filtered using a 70 *μ*m cell strainer. The filtrate was centrifuged at 800 rpm for 3 min and then resuspended with DMEM/F12 containing 10% FBS and 1% P/S. Cells were plated at a density of 2.5 × 10^4^ cells/cm^2^ in 75 cm^2^ flasks precoated with PLL and placed in a CO_2_ incubator in a 5% CO_2_ atmosphere at 37°C. After 6 days, astrocytes were used in coculture experiments as “fresh” astrocytes.

### 2.4. Isolation of BMECs

Primary BMECs were obtained as our previous study [17]. In total, BMECs were prepared from 2-week-old SD rats as previously described with modifications. The meninges and white matter were removed, and brain cortices were isolated. After centrifugation at 800 rpm for 3 min, the precipitate layer was added with an equal volume of 25% BSA and resuspended, and it was centrifuged 4 times at 3000 rpm for 5 min. The obtained microvessels were then digested in collagenase type II (1.0 mg/mL, Sigma-Aldrich) at 37°C for 1 h. The suspension was centrifuged at 800 rpm for 5 min and resuspended with DMEM/F12 with 20% FBS and 1% P/S. The BMECs were seeded on 75 cm^2^ plastic dishes precoated with 2% gelatin. The cultures were maintained in a 37°C incubator under humidified 5% CO_2_/95% air. The culture medium was changed every 2 days prior to use in the in vitro NVU model.

### 2.5. Immunofluorescence Staining and Image Acquisition

Cells were washed three times with 0.01 M phosphate-buffered saline (PBS) and fixed with 4% paraformaldehyde for 30 min. After the removal of excess paraformaldehyde, cells were blocked and permeabilized for 1 h using a mixture of 0.1% Triton X-100 (Fisher Scientific) and 10% goat serum (Sigma) in PBS. The cells were separately incubated overnight at 4°C with different primary antibodies; the primary antibodies for neuron, astrocyte, and BMEC detection were anti-microtubule associated protein 2 (MAP2, Abcam, Cambridge, UK), anti-glial fibrillary acidic protein (GFAP, Abcam), and anti-von Willebrand factor (VWf, Abcam), respectively. The tight junction protein was labeled with anti-zonula occludens-1 (ZO-1, Proteintech, Chicago, IL, USA) antibody overnight at 4°C. The following day, cells were incubated with secondary antibodies for 1 h (Alexa Fluor 647-conjugated goat anti-chicken IgY, Abcam; FITC-conjugated goat anti-mouse IgG, Abcam; Alexa Fluor 555-conjugated donkey anti-rabbit IgG, Abcam; and Alexa Fluor 488-conjugated goat anti-rabbit, Proteintech). Cells nuclei were stained with 4′,6-diamidino-2-phenylindole (Solarbio, Beijing, China) at a concentration of 0.5 *μ*g/mL for 10 min. Fluorescence images were captured with a fluorescence microscope (FluoView 1000, Olympus, Tokyo, Japan).

### 2.6. Establishment of the In Vitro NVU

**Figure 1:**
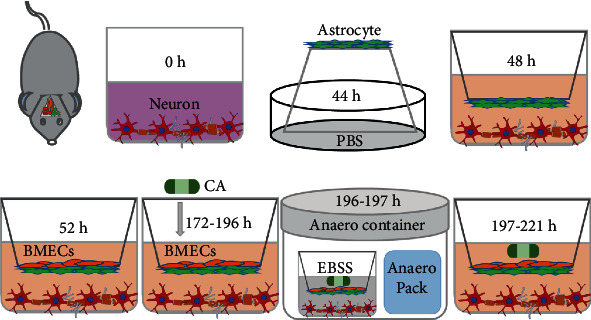
Experimental steps of this study. Neurons were cultured at the bottom of the Transwell filter at 0 h. After 44 h, astrocytes were seeded on the upper side of the inserts. After 52 h, BMECs were seeded on the inner side of the inserts. Experiments were performed after 120 hours of coculture. Based on the establishment of the in vitro NVU system, structural and functional evaluations under physiological conditions were undertaken at 172 h. The cells were treated with CA for 24 hours prior to OGD/R. To mimic ischemic conditions, cocultures were subjected to OGD for 1 h, and the cultures returned to the normoxic incubator for 24 h. The structural and functional evaluations under pathophysiological conditions were undertaken at 221 h.

Before starting the coculture, neurons were cultured on the bottom of Transwell plates (Corning, 3460, 0.4 *μ*m, New York, NY, USA), and the cultures were maintained in Neurobasal-A medium containing 2% B27, 0.25% GlutaMAX, and 1% P/S for at least 48 h. When the confluence gradually increased to 80%, astrocytes and BMECs were used to establish the model. Astrocytes (2 × 10^5^ cells/cm^2^) were seeded into the matching well under the insert membrane in the petri dish at 44 h. After 4 h, the Transwell insert was placed upside down in cell culture plates. BMECs were seeded on the inner side of the inserts coated with gelatin at a density of 3 × 10^5^ cells/cm^2^ in 0.5 mL medium at 52 h. The time when the neurons were plated was defined as zero in vitro. At 172 h, the experiments were performed. The procedure for establishing the model is shown in Figure 1. As controls, BMECs or neurons were also cultured alone on the Transwell chamber as group B and group N, respectively. BMECs were cultured with astrocytes or neurons as the B+A group and the B+N group, respectively. Neurons were cultured with astrocytes as the A+N group. Neurons were cultured with astrocytes and BMECs as the B+A+N group.

### 2.7. Sodium Fluorescein Permeability Measurements

We evaluated the permeability coefficient for small molecular (376 Da) sodium fluorescein. Sodium fluorescein was added in the upper chamber (at 100 *μ*g/mL), and 100 *μ*L aliquots were collected from the lower chamber to a microplate, and the volume was replaced with preequilibrated blank culture media at 15, 30, 52, and 67 min. The absorbances of the samples were measured using a fluorospectrophotometer (FLUOstar Omega, BMG LABTECH, Offenburg, Germany). Apparent permeability (Papp) of each group was calculated by applying Papp = (*dM*/*dt*)/(*A* × *C*), where *dM*/*dt* is the cumulative measured fluorescence intensity in the lower chamber per unit time corrected for dilution due to sampling, *A* is the surface area of the insert membrane (1.12 cm^2^), and *C* is the fluorescence intensity in the upper chamber.

### 2.8. Transendothelial Electrical Resistance Measurements

To characterize the formation of a tight endothelial cell monolayer, TEER was obtained using an epithelial volt-ohm resistance meter (ERS-2, Millipore, Germany) and the overall resistance to the current between electrodes was measured. The resistance value of the blank inserts treated with gelatin was subtracted from the total resistance measured. TEER values are expressed as *Ω* × cm^2^ by multiplying by the surface area of the Transwell insert.

### 2.9. OGD/R Insult on NVU and Drug Administration

As shown in Figure 1, to mimic ischemic conditions, OGD/R was used on the in vitro NVU model. Briefly, the medium was replaced with deoxygenated Earle's balanced salt solution (EBSS, Leagene Biotech Co., Beijing, China) at 196 h. Then, the NVU models were placed into the sealed Anaero container with an Anaero Pack (Mitsubishi, Tokyo, Japan) for 1 hour to initiate the OGD insult. OGD was terminated by complete medium, and the cocultures were cultured under normoxic conditions at 37°C for 24 hours to mimic reoxygenation. The cocultures with the above treatment were defined as the model group. The cocultures in the control group were incubated in DMEM/F12 with 20% FBS and 1% penicillin/streptomycin without the above OGD/R treatment.

CA was purchased from the National Institute for the Control of Pharmaceutical and Biological Products (Beijing, China). CA was dissolved in a cell culture medium with 10% NaOH to prepare the CA solution. The solution of CA (3.0 mg/mL) was prepared in deoxygenated glucose-free EBSS and DMEM/F12 containing 20% FBS and 1% P/S. The cultures were randomly divided into four groups: (1) control group; (2) model group; (3) CA-H group (93.75 *μ*g/mL); and (4) CA-L group (11.72 *μ*g/mL). The control group was incubated in complete medium. The cells in the CA-H group and CA-L group were treated with CA for 24 hours prior to OGD/R and then under OGD conditions for 1 h. OGD was terminated, and the cells were cultured for a further 24 h under normoxic conditions in the presence of CA. The cocultures in the CA-H and CA-L groups were treated with CA for 24 h before OGD/R and throughout the OGD/R process.

### 2.10. Flow Cytometric Analysis of Apoptosis

Analysis of apoptosis was carried out by flow cytometry using the Annexin V apoptosis detection kit. Cells were digested with EDTA-free enzymes, harvested and washed twice with PBS, then resuspended in 500 *μ*L binding buffer. Following this, cells were stained in 5 *μ*L Annexin V FITC and 5 *μ*L PI (KeyGen, Nanjing, Jiangsu Province, China), and the resuspended cells were incubated for 10 min in the dark. Finally, cells were immediately examined on a FACSCalibur (BD Biosciences, Franklin, NJ, USA) flow cytometer, and the data were analyzed with CellQuest software (BD Biosciences).

### 2.11. Assay for Inflammation, Neurotrophic Factor, and Oxidation Stress Activity

Interleukin-1*β* (IL-1*β*; Wuhan Liuhe Biotechnology Co., Wuhan, China), interleukin-6 (IL-6; Wuhan Liuhe Biotechnology Co), tumor necrosis factor-*α* (TNF-*α*; Proteintech), brain-derived neurotrophic factor (BDNF; Wuhan Liuhe Biotechnology Co.), and glial cell line-derived neurotrophic factor (GDNF; ayBiotech, Guangzhou, China) were assessed using ELISA kits according to the manufacturer's instructions. The optical density was measured at the wavelength of 450 nm using a microplate reader (BioTek, Winooski, Vermont, USA). Malondialdehyde (MDA; Jiancheng, Nanjing, China), superoxide dismutase (SOD; Jiancheng), lactate dehydrogenase (LDH; Jiancheng), and gamma-glutamyl transpeptidase (*γ*-GT; Jiancheng) were measured using commercial kits according to the manufacturer's instructions.

### 2.12. Western Blot (WB) Analysis

The changes in protein levels were quantified using WB analysis. Cells were washed with PBS. Proteins were obtained using the Minute™ Plasma Membrane Protein Isolation and Cell Fractionation Kit (Invent Biotechnologies) according to the manufacturer's instructions. The resuspended supernatant was quantified for the protein concentration using the BCA protein assay kit (KeyGen). Protein samples (30 mg per sample) were resolved using a 10% Tris/Glycine SDS-PAGE gel and then transferred to a polyvinylidene difluoride membrane. Membranes were incubated in PBST containing 5% nonfat milk for 30 min. Following incubation with the primary antibodies in PBST/TBST containing 5% BSA that recognize anti-cysteinyl aspartate specific proteinase-3 (caspase-3, Proteintech), anti-cysteinyl aspartate specific proteinase-9 (caspase-9, Proteintech), anti-B-cell lymphoma 2 (Bcl-2, Affinity), anti-Bcl-2-associated X protein (Bax, Proteintech), anti-ZO-1 (Proteintech), anti-claudin-5 (Affinity Biosciences, OH, USA), anti-occludin (Abcam), anti-TrkB (Affinity), anti-p-TrkB (Affinity), anti-cAMP response element-binding protein (anti-CREB, Affinity), anti-PI3K (p85, Affinity), anti-p-PI3K (p85, Affinity), anti-Akt (Ser473, Affinity), anti-p-Akt (Ser473, Affinity), anti-MAPK (p38, Affinity), anti-p-MAPK (p38, Affinity), anti-Erk1/2 (Affinity), anti-CREB (Affinity), anti-p-Erk1/2 (Affinity), and anti-GAPDH or anti-*β*-actin at 4°C overnight, the blots were washed and then incubated with anti-rabbit IgG (1 : 2000–1 : 4000) for 1 h at 25°C. After subsequent washes in TBST, the immunolabeling was detected using enhanced chemiluminescence reagents (PerkinElmer, Waltham, MA).

### 2.13. Statistics

All experiments were performed in triplicate or more. All data are expressed as mean ± standard deviation. Multiple comparisons were performed using one-way analysis of variance (ANOVA), and each group of data was subjected to the least significant difference (LSD) test using SPSS 20.0 (SPSS, Chicago, IL, USA). Values of *p* < 0.05 were considered to be statistically significant and *p* < 0.01 highly significant differences.

## 3. Results

### 3.1. The BBB Function and Neuronal Biological Behaviors in the NVU Model

**Figure 2:**
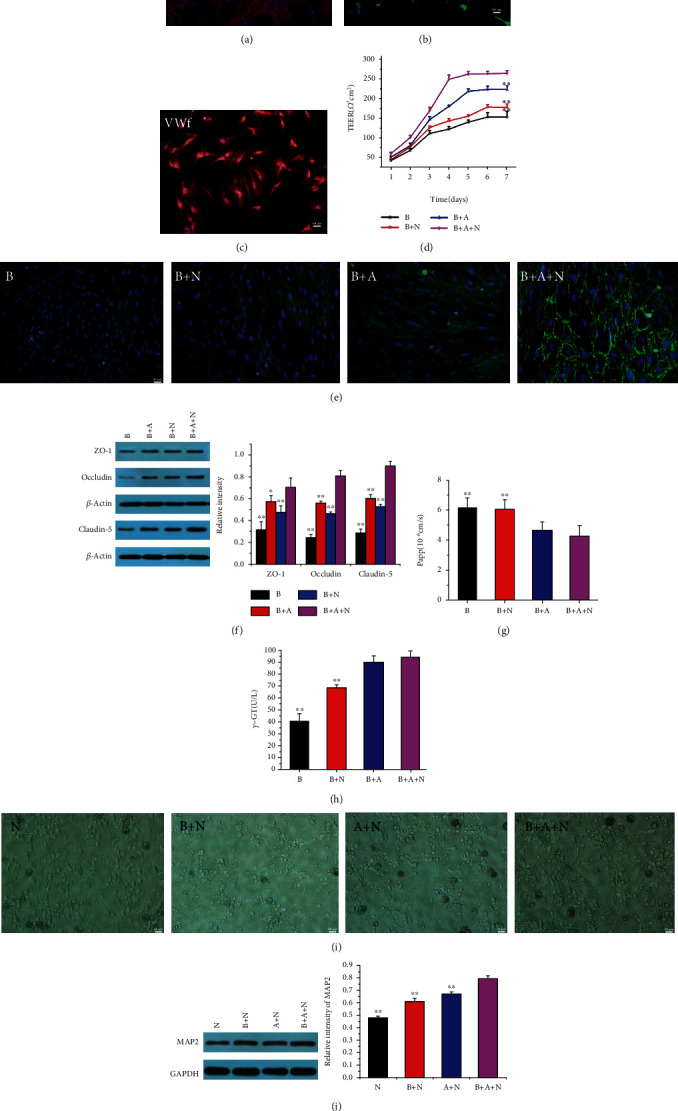
The BBB function and neuronal biological behaviors in the NVU model. (a) Neurons were positive for MAP2 (red). (b) Astrocytes were positive for GFAP (green). (c) BMECs were positive for VWf (red). (d) TEER was continuously tested within 7 d in BMECs: B group (B), B+N group (B+N), B+A group (B+A), and B+A+N group (B+A+N). (e) Immunostaining for ZO-1 (green, arrow) in B, B+N, B+A, and B+A+N. (f) Expression of tight junction protein (ZO-1, occludin, and claudin-5) in B, B+N, B+A, and B+A+N. (g) The permeability coefficient of SF in B, B+N, B+A, and B+A+N. (h) Expression of *γ*-GT in B, B+N, B+A, and B+A+N. (i) Neuronal morphology in N, B+N, A+N group (A+N), and B+A+N. (j) Expression of MAP2 in N, B+N, A+N, and B+A+N. Quantified results were normalized to *β*-actin expression. ^∗∗^*p* < 0.01 vs. the B+A+N group.

The purity of neurons, astrocytes, and BMECs was >93, >99, and 100%, respectively (Figures 2(a)–2(c)). We then established the in vitro NVU models with the above primary cells and examined BBB function and neuronal morphology. First, we assessed BBB function (Figures 2(d)–2(h)) in terms of tight junction protein expression, permeability, metabolizing enzyme activity, and electrical resistance. BMECs formed a monolayer at the cell boundaries and expressed ZO-1 by analysis of immunofluorescence. Immunofluorescence and western blotting results revealed that tight junction proteins were expressed when cultured with neurons and astrocytes at 120 h compared to single BMECs, and the expression was most abundant in the presence of astrocytes and neurons (Figures 2(e) and 2(f)). The feature of the in vitro BBB model was assessed by measuring the TEER or evaluating the permeability coefficient [24, 25]. We first used TEER as a measure of junctional tightness to detect the changes in TEER values due to different cocultures of BMECs with neurons and/or astrocytes. The triple cell coculture model yielded TEER that was significantly different from the monoculture control and coculture controls and reached a maximum TEER at 5 d (Figure 2(d)). As shown in Figure 2(g), the permeability coefficient of SF in the triple cell cocultures was the lowest compared with that in the monoculture of BMECs or BMECs cocultured with astrocytes or neurons. The *γ*-GT activity, which is a reliable marker for the BBB and abundantly present on the apical surface of endothelial cells [12], reached the highest value in the triple cell coculture models than other culture systems (Figure 2(h)). As shown in Figure 2(j), neurons acquired a more mature morphological phenotype in coculture with astrocytes and BMECs than other culture systems. MAP2, a specific marker of neuronal dendrites and cell bodies, was used to observe neurons for morphological changes in various culture systems [26]. WB results showed that the MAP2 level was significantly higher in the in vitro NVU model than in the monoculture, cocultured with BMECs or astrocytes (Figure 2(i)). Neuronal axons were fully extended and formed a distinct network in the triple cell coculture models. These results indicated that BMECs and astrocytes had a promoting effect on the neurite outgrowth, neuronal network formation, and the development of smooth, round cell bodies. We concluded that the in vitro NVU model was successfully established and had advantages over other culture systems. The in vitro NVU model was more suitable for understanding the functions and interactions of cells that make up the NVU.

### 3.2. The BBB Function and Neuronal Apoptosis in the NVU Exposed to OGD/R

**Figure 3:**
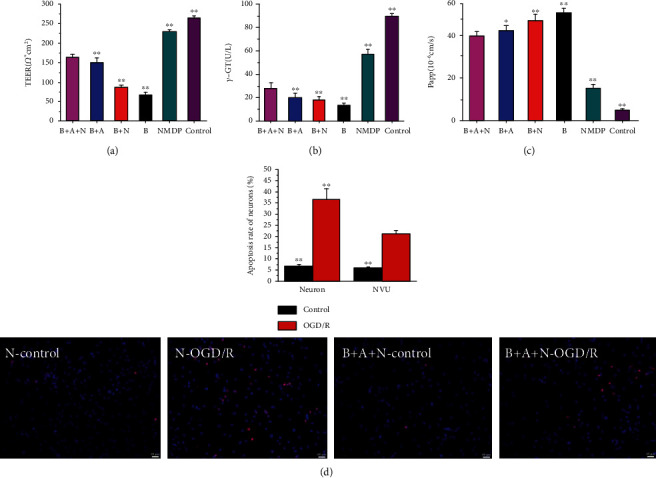
(a) TEER in B, B+N, B+A, and B+A+N. (b) *γ*-GT in B, B+N, B+A, and B+A+N. (c) Papp in B, B+N, B+A, and B+A+N. (d) Neuron apoptosis in N and B+A+N. ^∗∗^*p* < 0.01 vs. the B+A+N group.

In order to determine the advantages of the three-cell coculture system, we investigated the effect of OGD/R on the BBB function and neurons in various culture systems. After OGD/R, compared with the control group, the TEER value (Figure 3(a)) and *γ*-GT (Figure 3(b)) activity significantly decreased, while the permeability coefficient of SF (Figure 3(c)) significantly increased, indicating that the integrity of BBB was destroyed in various culture systems (Figures 3(a)–3(c)). However, compared with the coculture group with single cells and two types of brain cells, the NVU group had the highest TEER value and *γ*-GT activity and the lowest permeability coefficient of SF. Nimodipine treatment significantly reversed the changes in BBB function caused by OGD/R. Moreover, the results showed that the OGD/R induced significant neuronal apoptosis, and the number of apoptotic neurons in the B+A+N group was significantly less than that in the N group (Figure 3(d)). Significantly higher neuroprotection was detected in the NVU model in comparison to the monoculture model. Therefore, the in vitro NVU model was more suitable for evaluating the therapeutic effects of drugs on neurons than single cultured neurons commonly used in previous studies.

### 3.3. The Effects of CA on the In Vitro NVU Model after OGD/R

**Figure 4:**
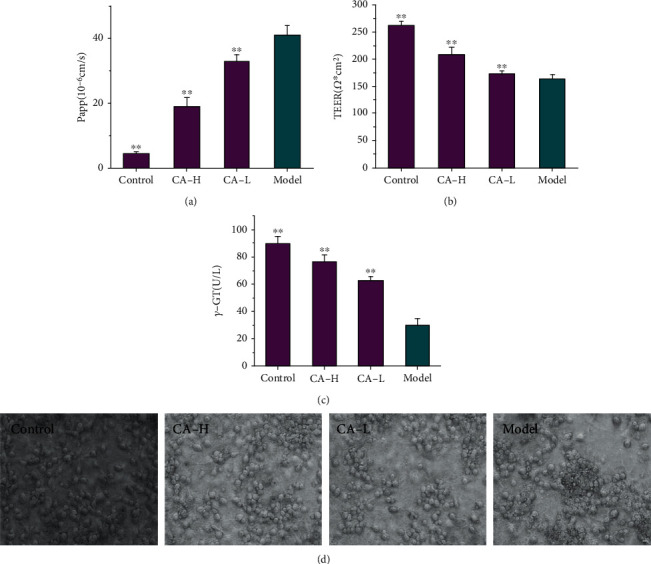
The effect of CA (93.75 *μ*g/mL, 11.72 *μ*g/mL) on in vitro NVU after OGD/R. (a) The effect of CA on Papp after OGD/R. (b) The effect of CA on TEER after OGD/R. (c) The effect of CA on *γ*-GT after OGD/R. (d) The effect of CA on neurons after OGD/R. ^∗∗^*p* < 0.01 vs. the model group.

As shown in Figures 4(a)–4(c), treatment with CA led to a decreased permeability coefficient of SF (Figure 4(a)) and increased TEER value (Figure 4(b)) and *γ*-GT activity (Figure 4(c)) compared with the model group. The results demonstrated that CA treatment improved the BBB function remarkably. Moreover, to detect the functional role of CA in regulating neuronal morphology, as shown in Figure 4(d), the data revealed that the number of neurons in the model field was much smaller than that in the control group, and cell bodies were disrupted, and neurites were shortened and thinned in the model group; CA had a significant protective effect on the injury neurites and neuronal cell bodies. CA has a significant protective effect on the BBB characteristics and neurons in the NVU after OGD/R.

### 3.4. The Effects of CA on Inflammation and Oxidative Damage after OGD/R in the In Vitro Model

**Figure 5:**
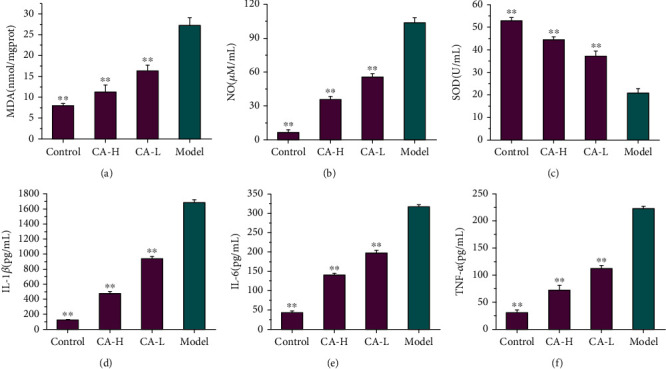
The effect of CA (93.75 *μ*g/mL, 11.72 *μ*g/mL) on inflammation and oxidative damage after OGD/R. (a) The effect of CA on MDA after OGD/R. (b) The effect of CA on NO after OGD/R. (c) The effect of CA on SOD after OGD/R. (d) The effect of CA on IL-1*β* after OGD/R. (e) The effect of CA on IL-6 after OGD/R. (f) The effect of CA on TNF-*α* after OGD/R. ^∗∗^*p* < 0.01 vs. the model group.

Accumulating evidence has shown that heightened oxidative stress is involved in the pathophysiology of IS [27]. As shown in Figures 5(a)–5(c), after anoxia-reoxygenation, MDA (Figure 5(a)) and NO (Figure 5(b)) significantly increased, and SOD (Figure 5(c)) significantly decreased compared with those in the control groups, which indicated that there was cell damage caused by free radicals, lipid peroxidation, and oxidative stress. After CA treatment, these four parameters were significantly improved, indicating the effectiveness of CA on cell injury. Moreover, previous studies demonstrated that the levels of inflammatory cytokines increased after IS [28]. We measured the effect of CA on the levels of inflammatory cytokines, e.g., TNF-*α*, IL-1*β,* and IL-6 in the present study. The results showed that the levels of IL-1*β* (Figure 5(d)), IL-6 (Figure 5(e)), and TNF-*α* (Figure 5(f)) were increased significantly after OGD/R, and the CA groups demonstrated significantly lower IL-1*β*, IL-6, and TNF-*α* levels compared with those in the model group. The release of inflammatory cytokines was inhibited through CA treatment.

### 3.5. The Effects of CA on Apoptosis after OGD/R

**Figure 6:**
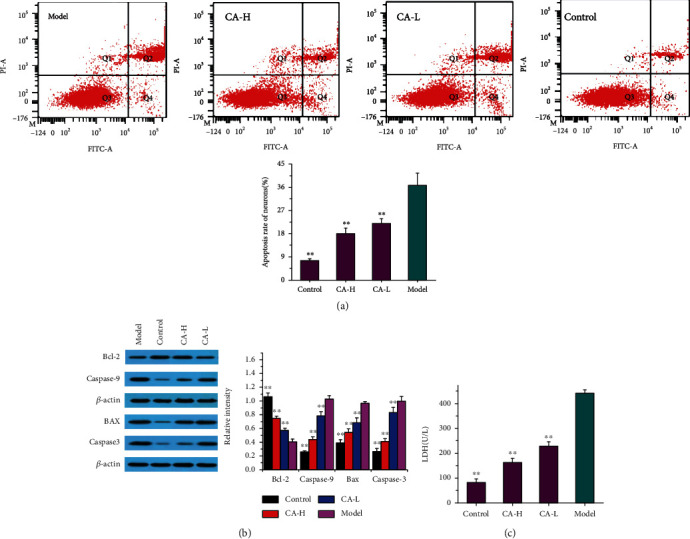
The effect of CA (93.75 *μ*g/mL, 11.72 *μ*g/mL) on apoptosis after OGD/R. (a) The effect of CA on apoptosis of neurons. (b) The effect of CA on the expression of Bcl-2, Bax, caspase-3, and caspase-9. (c) The effect of CA on the expression of LDH. Quantified results were normalized to *β*-actin expression. *p* < 0.01 vs. the model group.

The previous study indicated that neuronal apoptosis was involved in the occurrence of IS [29]. Flow cytometry was used to detect apoptosis of experimental groups for neurons, as shown in Figure 6(a). The results showed that the difference of the apoptosis percentages between the OGD/R group and the other groups was statistically significant, with the apoptosis percentages of the OGD/R group higher compared to those of the CA groups. CA pretreatment fully reversed OGD/R-induced cell apoptosis in neurons (Figure 6(a)). The expression of apoptotic proteins in the in vitro NVU systems was examined. As shown in Figure 6(b), Bcl-2 expression was significantly lower in the OGD/R group than in the control group, while the CA group demonstrated higher Bcl-2 expression than the OGD/R group. However, in contrast to Bcl-2 expression, expression of Bax, caspase-3, and caspase-9 was significantly higher in the model group than in the control group, while lower expression of Bax, caspase-3, and caspase-9 was observed in the treatment groups than in the model group. Moreover, we used diagnostic kits to measure the amount of LDH leakage release as an indicator of cell injury or death. After anoxia-reoxygenation, LDH (Figure 6(c)) significantly increased compared with those in the control groups, which indicated there was cell damage caused by inflammation, free radicals, lipid peroxidation, and oxidative stress. After CA treatment, LDH was significantly decreased, indicating the effectiveness of CA on cell injury and death.

### 3.6. CA Increases BDNF and Triggers TrkB/PI3K/Akt Signaling and TrkB/MAPK/Erk1/2 Signaling after OGD/R

**Figure 7:**
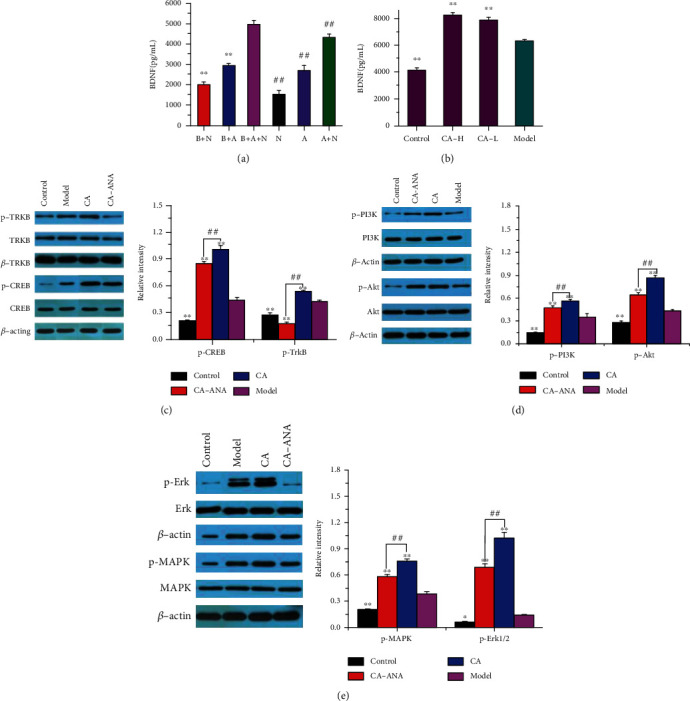
The effect of CA (93.75 *μ*g/mL, 11.72 *μ*g/mL) on BDNF and GDNF after OGD/R. (a) The level of BDNF in B+N, B+A, B+A+N, N, A, and A+N. (b) The effect of CA on the expression of BDNF. (c) The effect of CA on the expression of p-TrkB and p-CREB. (d) The effect of CA on the expression of p-PI3K and p-Akt. (e) The effect of CA on the expression of p-MAPK and p-Erk1/2. Quantified results were normalized to *β*-actin expression. (a) ^∗∗^*p* < 0.01 vs. the B+A+N group. (b–e) ^∗∗^*p* < 0.01 vs. the model group.

There is a wealth of evidence to support that BDNF are important contributors to protect neurons suffering from a stroke injury. Therefore, we examined the expression of BDNF and its specific receptor tropomyosin-related kinase B (TrkB), along with their downstream signaling mediated by MAPK/Erk1/2 and PI3K/Akt. The study found that the levels of BDNF (Figure 6(a)) were highest in the NVU model, and when astrocytes and neurons were cultured alone, the levels of BDNF were lower than those in coculture. Moreover, we found that BDNF (Figure 6(b)) were synthesized more in the model group than in the control group, and CA treatment further promoted this progress. In the current study, TrkB proteins (Figure 7(c)) are significantly increased by OGD/R compared with those of the control, while CA can increase more expressions of TrkB based on OGD/R. In addition, we found that CA treatment upregulated CREB (Figure 7(c)), PI3K (Figure 7(d)), Akt (Figure 7(d)), MAPK (Figure 7(e)), and Erk (Figure 7(e)) phosphorylation. Moreover, as an antagonist of TrkB receptors, ANA12 was used to block TrkB activation in CA-treated NVU systems. However, these positive effects on the downstream proteins of BDNF were suppressed by the treatment of ANA12.

## 4. Discussion

Neurons, astrocytes, and microvascular endothelial cells are the main components of NVU and play multiple roles in actively regulating vascular and neuronal functions. Interestingly, comparing monocultures, BMECs, and BMECs grown with neurons and astrocytes in the lower compartment showed significantly enhanced barrier integrity. In addition, BMECs and astrocytes had a remarkable promoting effect on inducing a significant enhancement of neurite outgrowth and neuronal network formation and smooth, round cell bodies. The promotion effect caused by astrocytes is more obvious. As numerous cells in the mammalian brain, astrocytes play many important roles in maintaining normal brain function, including structural support, BBB formation and maintenance, neuronal metabolism, maintenance of the extracellular environment, regulation of cerebral blood flow, stabilization of cellular communication, neurotransmitter synthesis, and antioxidative stress defense [30–32]. Therefore, astrocytes also play an important role in NVU. (1) Astrocytes play metabolic and structural support roles and contribute to the integrity of the BBB, linking communication between neurons and the endothelium [33, 34]. (2) Astrocytes have been clearly shown to assist in improving the tightness of BMECs and inducing proteins in the cell membrane, which can be used to establish an in vitro BBB model and replicate SD brain internality. (3) Astrocytes promote neuronal growth, differentiation, maturation, axonal growth, connection, and maintenance and maintain the microenvironment of neurons, neuronal plasticity, survival, and function [33, 35]. This suggests that astrocytes affect the neurons and BBB maturation in a different fashion. In addition, compared with single cultured neurons or BMECs, NVU is more able to resist OGD/R-induced injury. In summary, compared with BMECs or neurons only, the NVU with more mature BBB and neurons, as a complete structural and functional unit, is more representative of the integrity of cerebral function.

To our knowledge, the present study is the first to show that the neuroprotective effects of CA in the ischemic brain are due to its ability to preserve NVU integrity by stabilizing the BBB and protecting neurons, using an OGD/R model of IS. CA protects BBB and neurons from ischemic injury by reducing the level of overall NVU apoptosis, oxidative stress, inflammation, and neuronal loss. CA as one of the components of bile acid has been reported to be able to diffuse in the phospholipid bilayer and further cross the blood-brain barrier [18]. Also, as the main active ingredient of Chinese medicine bezoar, CA has been proved to reduce inflammatory injury and have antiapoptotic and anti-inflammatory effects [19, 20]. Moreover, CA protects rat BMECs from injury induced by an in vitro ischemia-reperfusion [36]. More importantly, we found that BDNF is most abundant in NVU and is mainly released by astrocytes after OGD/R, and treatment with CA remarkably increased the expression of BDNF compared to the model group. BDNF is essential for neuronal growth, maturation, and maintenance, as well as for neuronal plasticity, participates in axonal and dendritic differentiation, and enhances neuronal cell survival [37, 38]. Previous experimental and clinical studies have indicated that BDNF is involved in repair mechanisms after ischemic injury [39]. BDNF improves survival and neuroprotective functions via binding to TrkB to form a complex [23]. The downstream signaling elicited by the BDNF-TrkB complex has many of the characteristic features of receptor tyrosine kinases, including activation of the MAPK/Erk and PI3K/Akt pathways [22]. In the current study, we found that CA treatment upregulates CREB, PI3K, Akt, MAPK, and Erk phosphorylation. The results were confirmed by the application of ANA12. Inhibition of the PI3K/Akt pathway and MAPK/Erk pathway by ANA12 reversed the increase of p-PI3K, p-Akt, p-MAPK, and p-Erk levels. The PI3K/Akt pathway is particularly imperative for mediating antiapoptotic and prosurvival activity and enhancing dendritic growth and branching in a wide range of ways [23]. The MAPK/Erk pathway is important for mediating neuronal survival and promoting neuronal differentiation and survival under a wide variety of circumstances [23, 40]. The PI3K/Akt and MAPK/Erk signaling pathways are involved in proliferation, oxidation stress, inflammation, and apoptosis after ischemic stroke [22, 41]. The important transcription factor that mediates BDNF transcriptional regulation is the cAMP BDNF transcriptional regulation is the CREB, which is necessary for BDNF to induce dendritic branching of neurons and regeneration of damaged neurons [42]. Our data provides evidence that CA may exert neuroprotective effects through the BDNF-TrkB-PI3K/Akt pathway and BDNF-TrkB-MAPK/Erk pathway. Taken together, CA works by modulating neuroinflammation, oxidative damage, and growth factors, especially by triggering BDNF-TrkB-MAPK/Erk and BDNF-TrkB-PI3K/Akt signaling, eventually leading to the recovery of BBB function and neuron phenotype in NVU. Significant improvement in BBB integrity and neuron survival in NVU suggests a stronger therapeutic potential of CA for human IS patients in the future.

## 5. Conclusion

NVU is considered to be a more comprehensive target for the treatment of IS. We successfully established an in vitro NVU and performed further identification of a previous study. The in vitro NVU model was more suitable for understanding the functions and interactions of cells and the protective effect of drugs on multiple cells. The study indicated that CA works by modulating neuroinflammation, oxidative damage, and growth factors eventually leading to the recovery of BBB function and neuron phenotype in NVU. CA may exert neuroprotective effects through the BDNF-TrkB-PI3K/Akt pathway and BDNF-TrkB-MAPK/Erk pathway. Therefore, CA possesses the effect of antagonizing multiple mechanisms of ischemic stroke and protecting multiple cell types in NVU and may be useful as a treatment for IS.

## Data Availability

The data used to support the findings of this study are available from the corresponding authors upon request.
